# Editorial: Advances in Intravascular Imaging

**DOI:** 10.3389/fcvm.2021.764378

**Published:** 2021-09-16

**Authors:** Christos V. Bourantas, Farouc A. Jaffer, Yoshinubo Onuma, Patrick W. Serruys

**Affiliations:** ^1^Department of Cardiology, Barts Heart Centre, Barts Health NHS, London, United Kingdom; ^2^Institute of Cardiovascular Sciences, University College London, London, United Kingdom; ^3^William Harvey Research Institute, Queen Mary University London, London, United Kingdom; ^4^Cardiovascular Research Center and Cardiology Division, Harvard Medical School and Massachusetts General Hospital, Boston, MA, United States; ^5^Department of Cardiology, National University of Ireland, Galway, Ireland; ^6^Faculty of Medicine, National Heart & Lung Institute, Imperial College London, London, United Kingdom

**Keywords:** hybrid intravascular imaging, coronary artery disease, percutaneous coronary intervention, intravascular ultrasound, optical coherence tomography

Intravascular imaging was introduced 30 years ago to assess coronary artery morphology and facilitate percutaneous coronary intervention (PCI). Today, invasive imaging has an established role in clinical practice and research (1). Cumulative data over the recent years have underscored the value of intravascular ultrasound (IVUS) and optical coherence tomography (OCT) in optimizing PCI results, while prospective studies of coronary atherosclerosis have provided convincing evidence that both modalities are capable to detect lesions that are likely to progress and cause events. These reports however, also revealed limitations of these techniques in guiding PCI and in identifying vulnerable plaques (2). To overcome these drawbacks efforts have been made to design advanced intravascular imaging systems that can provide a more detailed and complete evaluation of plaque pathology and biology, either by combining two intravascular imaging probes or by enabling acquisition of additional information by a single imaging probe (3). This special issue of Frontiers in Cardiovascular Medicine provides an overview of the developments in the field and summarizes the evidence supporting the use of novel intravascular imaging catheters in clinical practice and research.

Near-infrared spectroscopy (NIRS)-IVUS imaging is the first hybrid intravascular imaging modality that had clinical applications. The catheter incorporates a NIRS and IVUS integrated probe that acquire simultaneous imaging data which are co-registered to generate hybrid NIRS-IVUS images. The review article of Kuku et al. published in this issue describes the evolution in catheter design and presents the evidence supporting the value of this modality in guiding PCI and detecting vulnerable plaques. Muller—the inventor of NIRS imaging—and Madder, also highlight, in their review, the potential of hybrid NIRS-based imaging in optimizing PCI results and identifying high-risk lesions and patients who would benefit from aggressive treatments targeting atherosclerosis, and introduce a combined NIRS-OCT system that is expected to have clinical applications in the near future (Muller and Madder). Another hybrid intravascular imaging modality that appears superior to standalone IVUS or OCT imaging in assessing plaque phenotype is the combined IVUS-OCT imaging, which has also reached clinical translation. Several prototypes have been presented over the last years and one of them the Novasight Hybrid^TM^ System (Conavi Medical Inc, Toronto, Canada) had recently first-in-man studies (4). Ono et al. in their review discuss the challenges of combined IVUS-OCT imaging, present the evidence supporting its use in PCI and summarize the findings of histology studies which indicate that hybrid IVUS-OCT imaging is superior to standalone IVUS and OCT in assessing plaque features associated with increased vulnerability (4).

The above developments in intravascular imaging may enable more precise evaluation of plaque composition but cannot detect vascular inflammation which is an important instigator of plaque evolution. Near-infrared fluorescence (NIRF) imaging was introduced to address this unmet need. This modality can be combined with IVUS or OCT and appears capable to identify the presence of macrophages, cathepsin protease activity, oxidized low-density lipoprotein and thrombus. NIR autofluorescence (NIRAF)-OCT intracoronary imaging has been performed in patients and may identify high-risk plaques with intraplaque hemorrhage or oxidized lipid, without the need for a contrast agent. The developments in the field are described in detail by the inventors of NIRF in a comprehensive review that also discusses the potential value of hybrid NIRF-IVUS and OCT-NIRF imaging in secondary prevention (Khraishah and Jaffer).

Another intravascular imaging modality that is capable to accurately detect the presence of macrophages in the superficial plaque and thus vascular biology is florescence lifetime imaging (FLIm). The study of Bec et al. published in this special issue of Frontiers in Cardiovascular Medicine compared the efficacy of FLIm and Raman spectroscopy in characterizing plaque composition using histology as reference standard. The authors showed that FLIm, in contrast to Raman spectroscopy, has a limited efficacy in detecting different tissue types. In this report the co-registered FLIm and Raman data allowed more precise evaluation of the efficacy of FLIm imaging in characterizing plaque composition and showed that increased lifetime values are associated with the presence of cholesterol and carotenes and not with collagen, as it was shown in previous studies.

In parallel to the design of novel hybrid imaging catheters for more detailed assessment of the atheroma, efforts have been made to optimize standalone intravascular imaging catheters. Toward this direction polarization sensitive (PS)-OCT imaging was introduced. This approach relies on the analysis of the polarization properties of the reflected OCT signal (Otsuka et al.). The inventors of this approach present a thorough review which describes the basic principles of PS-OCT and highlights its value in characterizing atheroma. Summarizing their research in the field the authors suggest that PS-OCT is capable to assess collagen and smooth muscle cell content in fibrous caps, and improve the efficacy of OCT in identifying necrotic cores, macrophage accumulations and cholesterol crystals—plaque features that are seen in high-risk lesions. Finally, PS-OCT offers a unique potential to automatically quantify tissue types allowing fast processing of large imaging data.

Despite the high resolution of OCT there are plaque micro-features that cannot be visualized by this imaging technique. To address this limitation micro-(μ)OCT has been developed that allows plaque imaging with a resolution of 1–2 μm. Nishimiya and Tearney in a comprehensive review present the technical developments in the field, and highlight the potential of this approach in assessing plaque vulnerability and PCI results. Another emerging imaging modality is optically generated ultrasound imaging (OpUS). This approach offers increase signal penetration (>20 mm) and is capable to acquire images with a resolution of >50 μm. Preliminary data has shown that it is able to characterize plaque composition. OpUS is still in its infancy and its full potential has not been evaluated yet; moreover further developments are needed before this modality has applications in interventional cardiology (Little et al.).

From the above it is apparent that the field of intravascular imaging is rapidly evolving and that soon the interventional cardiologists will have numerous options for assessing plaque morphology and guiding percutaneous revascularisation. Which modality is likely to dominate in the clinical arena is difficult to predict, as this will depend not only on its imaging capabilities but also on the time needed to interrogate a vessel, on how easy will be image interpretation and on how fast will be data post-processing. What is obvious however, is that the emerging imaging techniques are likely to fulfill our expectations allowing not only better treatment planning but also complete visualization of vessel pathology, biology and physiology—after coronary reconstruction and blood flow simulation with easy to use software (Kilic et al.)—and thus more precise identification of vulnerable lesions and high-risks patients that will benefit from emerging therapies targeting atherosclerosis (5).

In addition, the above advances in intravascular imaging is also likely to enable more precise evaluation of new developments in non-invasive imaging and in particular in computed tomography coronary angiography (CTCA) and positron emission tomography (PET)-CT/CTCA. In contrast to intravascular imaging that is associated with a risk of complications and do not allow complete assessment of the coronary artery tree, non-invasive imaging appears as the ideal approach for stratifying cardiovascular risk as it can be used in asymptomatic individuals, requires a lower dose of radiation and enables complete study of all the major coronary arteries (5). Yet CTCA and PET-CT/CTCA image analysis is time consuming, the positive predictive value of CTCA in detecting vulnerable plaques is low while the additive value of PET-CT/CTCA to stratify cardiovascular risk over CTCA has not been well-studied (6). Therefore, further research is needed, and developments are required to enhance image resolution expedite data analysis and identify new imaging-markers that will allow more precise identification of lesions at risk and enable the broad use of non-invasive imaging in the clinical arena to detect vulnerable patients that will benefit from emerging therapies targeting atherosclerosis.

## Author Contributions

CB drafted the manuscript while FJ, YO, and PS reviewed, edited, and approved the final draft. All authors contributed to the article and approved the submitted version.

## Conflict of Interest

The authors declare that the research was conducted in the absence of any commercial or financial relationships that could be construed as a potential conflict of interest.

## Publisher's Note

All claims expressed in this article are solely those of the authors and do not necessarily represent those of their affiliated organizations, or those of the publisher, the editors and the reviewers. Any product that may be evaluated in this article, or claim that may be made by its manufacturer, is not guaranteed or endorsed by the publisher.

## References

[1] Bourantas CV Tenekecioglu E Radu M Raber L Serruys PW. State of the art: role of intravascular imaging in the evolution of percutaneous coronary intervention - a 30-year review. EuroIntervention. (2017) 13:644–53. 10.4244/EIJ-D-17-00471 28844027

[2] Ramasamy A Serruys PW Jones DA Johnson TW Torii R Madden SP. Reliable *in vivo* intravascular imaging plaque characterization: a challenge unmet. Am Heart J. (2019) 218:20–31. 10.1016/j.ahj.2019.07.008 31655414

[3] Bourantas CV Garcia-Garcia HM Naka KK Sakellarios A Athanasiou L Fotiadis DI. Hybrid intravascular imaging: current applications and prospective potential in the study of coronary atherosclerosis. J Am Coll Cardiol. (2013) 61:1369–78. 10.1016/j.jacc.2012.10.057 23500282

[4] Ono M Kawashima H Hara H Gao C Wang R Kogame N. Advances in IVUS/OCT and future clinical perspective of novel hybrid catheter system in coronary imaging. Front Cardiovasc Med. (2020) 7:119. 10.3389/fcvm.2020.00119 32850981 PMC7411139

[5] Bourantas CV Garcia-Garcia HM Torii R Zhang Y-J Westwood M Crake T. Vulnerable plaque detection: an unrealistic quest or a feasible objective with a clinical value? Heart. (2016) 102:581–9. 10.1136/heartjnl-2015-309060 26783236

[6] Johnson TW Raber L di Mario C Bourantas C Jia H Mattesini A. Clinical use of intracoronary imaging. Part 2: acute coronary syndromes, ambiguous coronary angiography findings, and guiding interventional decision-making: an expert consensus document of the European Association of Percutaneous Cardiovascular Interventions. Eur Heart J. (2019) 40:2566–84. 10.1093/eurheartj/ehz332 31112213

